# Aplicação do Mapeamento Eletrocardiográfico de Superfície por Meio de Exercício em Pequenos Animais

**DOI:** 10.36660/abc.20220646

**Published:** 2022-11-01

**Authors:** Carlos Alberto Pastore

**Affiliations:** 1 Universidade de São Paulo Faculdade de Medicina Hospital das Clinicas São Paulo SP Brasil Instituto do Coração (InCor), Hospital das Clinicas FMUSP, Faculdade de Medicina, Universidade de São Paulo – Unidade Clínica de Eletrocardiografia de Repouso, São Paulo, SP – Brasil

**Keywords:** Exercício, Condicionamento Físico Animal, Mapeamento Potencial de Superfície Corporal, Miocárdio, Ratos, Ventrículos do Coração

Há mais de 100 anos, mapas registrando os potenciais elétricos do coração tentam representar a distribuição, na superfície do tórax, desses eventos elétricos internos. Os registros, em seu início com Waller, tentaram definir um vetor resultante de uma fonte bipolar, o “vetor do coração”, presumindo essa distribuição de potenciais como se um bipolo elétrico fosse colocado dentro do tórax e aplicado à superfície corporal, com um terceiro eletrodo adicionando o componente sagital do vetor. Os três seriam suficientes para fornecer toda a informação do eletrocardiograma (ECG) a ser extraída das medições da superfície corporal. Somente nas décadas de 30 e 40 procurou-se aumentar o número de eletrodos no tórax para detectar eventos ocorrendo em regiões cardíacas próximas às derivações precordiais. Após 1950, finalmente, estudos demonstraram que a complexidade das informações elétricas geradas no interior do coração era muito maior que as geradas por um bipolo único, com múltiplas frentes de ondas nos ventrículos criando correntes que fluem para fora e para dentro do coração em locais diversos; a distribuição dos potenciais exibiria, portanto, máximas e mínimas variando no tempo, localizadas geralmente em áreas não exploradas pelo eletrocardiograma convencional.

Novos métodos complementando o ECG e o vetorcardiograma (VCG) adicionaram mais eletrodos (dorso e precordial direito), e a partir de Wilson, o ECG de 12 derivações passou a contar com três eletrodos bipolares e três unipolares modificados no plano frontal, além de seis unipolares no tórax anterior (precordiais). Esses são suficientes para registrar a maior parte das informações sobre os eventos elétricos no coração. Porém, a expressão global da atividade elétrica miocárdica não pode ser captada sem que haja um número maior de derivações registradas simultaneamente.

O Mapeamento Eletrocardiográfico de Superfície *(BSPM, body surface potential mapping)* tem a possibilidade de detalhar espacialmente, de forma não invasiva, os componentes elétricos não bipolares, além do componente bipolar da atividade elétrica do coração. É sensível aos eventos regionais dentro do coração, pois capta a distribuição potencial na superfície corpórea e permite avaliar os vários aspectos do campo cardíaco.

Avaliar manualmente um grande número de eletrogramas registrados ao mesmo tempo exige processamento computadorizado, razão pela qual somente a partir da década de 60, com a evolução da informática, a técnica de BSPM tornou-se utilizável na prática. Nas décadas de 70 e 80, foram desenvolvidos vários sistemas de colocação de eletrodos torácicos.

A experiência do BSPM no Brasil teve início em 1990,^1–3^ quando conseguimos adquirir o equipamento da Fukuda Denshi 7100, o primeiro a ser fabricado no mundo e para uso do qual realizamos treinamento no Japão (Tóquio).

Desta forma, desenvolvemos, nestas três últimas décadas, trabalhos pioneiros em todas as especialidades da Cardiologia, nas quais tivemos a parceria no desenvolvimento de pesquisas e teses publicadas de diversos colegas do InCor.^4–13^

A Bioengenharia do InCor-HCFMUSP tem trabalhado no desenvolvimento de um sistema BSPM com 64 derivações totalmente nacional desde 2016, para o qual a Unidade de Eletrocardiografia de Repouso tem contribuído.

Nessa construção já foram aprovadas e publicadas as primeiras experiências na análise do BSPM, incluindo vetorcardiografia.^14,15^

A evolução da utilização do BSPM tem sido muito promissora, principalmente do ponto de vista experimental, pois agrega a possibilidade de colocar um número muito maior de derivações que o ECG convencional e uma avaliação de mapas isopotenciais e isócronos.

Nesta publicação do grupo russo liderado pela Dra. Irina Roshchevskaya,^16^ muito reconhecida em trabalhos experimentais com pequenos animais na Eletrocardiografia, encontramos experiências interessantes.

A primeira delas é a utilização do BSPM em pequenos animais, cuja adaptação dos eletrodos é um grande desafio, trazendo uma experiência inovadora neste campo.

Os autores avaliaram a atividade elétrica do coração durante a despolarização ventricular após exercícios exaustivos agudos em esteira, em ratos não treinados, utilizando o BSPM em combinação com o ECG convencional.

A utilização dos mapas isopotenciais, através do BSPM, para avaliar a atividade elétrica do coração durante a despolarização ventricular é uma ferramenta bem estudada. Neste estudo, a originalidade está no uso deste recurso em ratos submetidos a exercício físico exaustivo. A avaliação do BSPM, nesses animais submetidos ao exercício exaustivo, não mostrou alteração do padrão espacial das distribuições dos potenciais de superfície corporal durante a despolarização ventricular. Houve, sim, uma diminuição da duração da fase média e na duração total da despolarização ventricular, bem como mudanças na amplitude do extremo negativo dos BSPMs.

Os resultados mostraram que esta atividade causa alterações reversíveis nas características temporais e de amplitude do BSPM durante a despolarização ventricular, provavelmente relacionadas a alterações na excitação da massa principal do miocárdio ventricular. Estes achados podem contribuir para a análise da atividade elétrica cardíaca em atletas de alta performance.
