# Supplementary material for: Aplicação do Mapeamento Eletrocardiográfico de Superfície por Meio de Exercício em Pequenos Animais
Source: Arq Bras Cardiol. 2022 Nov 1;119(5):776–7. [Article in Portuguese] doi: 10.36660/abc.20220646 (PMC9750226; doi:10.36660/abc.20220646)
Supplement: Translation

# Translation: Applicability of Body Surface Potential Mapping Through Exercise in Small Animals

**Authors:** Carlos Alberto Pastore

**Affiliations:** 1 São Paulo SP Brazil Instituto do Coração (InCor), Hospital das Clinicas FMUSP, Faculdade de Medicina, Universidade de São Paulo – Unidade Clínica de Eletrocardiografia de Repouso, São Paulo, SP – Brazil

**Keywords:** Exercise, Physical Conditionning, Animal, Body Surface Potential Mapping, Myocardial, Rats, Heart Ventricle

The electrical potentials of the heart have been recorded for over a hundred years to represent the distribution of these internal electrical events on the torso surface. Such recordings that started with Waller tried to establish a resultant vector from a bipolar source, the “heart vector,” assuming that this distribution of potentials would act as if an electrical bipole was inserted inside the thorax and applied to the body surface, with a third electrode adding the sagittal component of the vector. The three would be enough to supply all the electrocardiogram (ECG) information extracted from measurements from the body surface. It was not until the 1930s and 1940s that the number of electrodes on the chest surface was tentatively increased to detect events occurring in cardiac areas near the precordial leads. Finally, after 1950 some studies demonstrated that the complexity of the electrical information generated inside the heart was far greater than that generated by a sole bipole, with multiple wavefronts in the ventricles creating currents that flow into and from the heart in several places; the potentials, therefore, would exhibit maximum and minimum distributions varying with time, usually located in areas unexplored by the conventional electrocardiogram.

New methodologies complementing the ECG and vectorcardiogram (ECG) added new electrodes (dorsal and right precordial leads), and, since Wilson, the 12-lead ECG started to have three bipolar and three unipolar modified leads in the frontal plane, in addition to six unipolar (precordial) leads on the anterior chest, which can record most of the information about electrical events in the heart. However, the myocardial electrical activity global expression cannot be captured without a larger number of simultaneously recorded leads.

The body surface potential mapping (BSPM) can provide, spatially and noninvasively, in great detail, both the electrical non-bipolar components and the bipolar component of the cardiac electrical activity. It is responsive to regional events inside the heart by capturing the distribution of potentials on the body surface and evaluating the various aspects within the cardiac field.

Manual assessment of a large number of electrograms recorded simultaneously demands computerized processing, and for this reason, it was only in the 1960s that the BSPM became usable in medical practice. Several systems were developed in the 1970s and 1980s to deploy electrodes across the torso.

In Brazil, the experience with the BSPM methodology began early in the 1990s,^1–3^ when we could acquire the 87-lead Fukuda Denshi 7100 equipment, the first to be manufactured in the world; we were trained for its use at the manufacturer's premises in Japan (Tokyo).

During the last three decades, thereby, we developed pioneering studies in all the specialties of Cardiology, including our partnership with colleagues developing doctoral theses at InCor HCFMUSP.^4–13^

The Biomedical Engineering Department of InCor HCFMUSP has been working on the development of a fully nationalized 64-lead BSPM equipment since 2016, with the contribution of the Electrocardiography Unit of InCor.

The first experiences with the BSPM analysis with this new equipment, including vectorcardiography, have already been approved and published.^14,15^

The evolution of BSPM utilization has been very promising, especially from the experimental viewpoint, since it enables placing a much bigger number of electrodes than with standard ECG and forward evaluation of isopotential and isochronal maps.

In the article by the Russian group headed by Dr. Irina Roshchevskaya,^16^ who has deep knowledge in experimental work with Electrocardiography in small animals, we find interesting experiences, such as using BSPM in small animals with a very challenging adaptation of electrodes; this work brings us an innovative experience in this field.

The authors evaluated the electrical activity of the heart of untrained rats during ventricular depolarization after the performance of acute exhaustive exercises on a treadmill using the BSPM combined with the standard ECG.

Using isopotential maps obtained from the BSPM to assess the heart's electrical activity during ventricular depolarization is an extensively studied tool. The novelty of this study is the use of this resource in rats submitted to exhaustive physical exercise. BSPM evaluation of these animals under such conditions did not reveal any alteration of the spatial pattern of body surface potential distribution during ventricular depolarization. What could be seen was a decrease in the duration of the middle phase and in the overall duration of the ventricular depolarization, as well as in the amplitude of BSPM negative extremum.

The results showed that this activity causes reversible alterations of BSPM temporal features and amplitudes during ventricular depolarization, which are related to alterations of the main ventricular myocardial mass excitability. These findings may contribute to the analysis of cardiac electrical activation in high-performance athletes.
